# Rapid combinatorial rewiring of metabolic networks for enhanced poly(3-hydroxybutyrate) production in *Corynebacterium glutamicum*

**DOI:** 10.1186/s12934-023-02037-x

**Published:** 2023-02-17

**Authors:** Sung Sun Yim, Jae Woong Choi, Yong Jae Lee, Ki Jun Jeong

**Affiliations:** 1 Department of Biological Sciences, KAIST, Daejeon, Republic of Korea; 2 Traditional Food Research Group, Korea Food Research Institute, Jeonju, Republic of Korea; 3 Cell Factory Research Center, Korea Research Institute of Bioscience and Biotechnology (KRIBB), Daejeon, 34141 Korea; 4 Department of Chemical and Biomolecular Engineering, KAIST, Daejeon, Republic of Korea; 5 Institute for BioCentury, KAIST, Daejeon, Republic of Korea; 6 Major of Environmental Biotechnology, KRIBB School of Biotechnology, Korea University of Science and Technology (UST), Daejeon, Korea

**Keywords:** *Corynebacterium glutamicum*, Metabolic engineering, Combinatorial optimization, Poly(3-hydroxybutyrate)

## Abstract

**Background:**

The disposal of plastic waste is a major environmental challenge. With recent advances in microbial genetic and metabolic engineering technologies, microbial polyhydroxyalkanoates (PHAs) are being used as next-generation biomaterials to replace petroleum-based synthetic plastics in a sustainable future. However, the relatively high production cost of bioprocesses hinders the production and application of microbial PHAs on an industrial scale.

**Results:**

Here, we describe a rapid strategy to rewire metabolic networks in an industrial microorganism, *Corynebacterium glutamicum*, for the enhanced production of poly(3-hydroxybutyrate) (PHB). A three-gene PHB biosynthetic pathway in *Rasltonia eutropha* was refactored for high-level gene expression. A fluorescence-based quantification assay for cellular PHB content using BODIPY was devised for the rapid fluorescence-activated cell sorting (FACS)-based screening of a large combinatorial metabolic network library constructed in *C. glutamicum*. Rewiring metabolic networks across the central carbon metabolism enabled highly efficient production of PHB up to 29% of dry cell weight with the highest cellular PHB productivity ever reported in *C. glutamicum* using a sole carbon source.

**Conclusions:**

We successfully constructed a heterologous PHB biosynthetic pathway and rapidly optimized metabolic networks across central metabolism in *C. glutamicum* for enhanced production of PHB using glucose or fructose as a sole carbon source in minimal media. We expect that this FACS-based metabolic rewiring framework will accelerate strain engineering processes for the production of diverse biochemicals and biopolymers.

**Supplementary Information:**

The online version contains supplementary material available at 10.1186/s12934-023-02037-x.

## Introduction

Plastics have become an integral part of our lives because of their numerous advantageous attributes, such as light weight, durability, and longevity. Hundreds of millions of tons of plastics are synthesized annually. More than 50% of plastics are not recycled, and are discarded directly into surrounding environments after a single use. This is broadly and detrimentally affecting ecosystems and human health [1]. Plastic pollution is one of the greatest challenges we face this century. On the other hand, bioplastics are alternative plastic materials that are produced from renewable bioresources.

Among many different types of bioplastics, polyhydroxyalkanoates (PHAs) are linear polyesters produced by microorganisms by fermentation of sugar or lipids [2, 3]. PHAs store carbon and energy in bacteria in the presence of excess carbon sources in their environment. PHAs are naturally biocompatible and biodegradable [4, 5]. Furthermore, their material properties can be modulated using many different types of monomers [6, 7], offering the capacity to replace many of the current petroleum-based plastics in a sustainable future [8]. Poly(3-hydroxybutyrate) (PHB) is the most frequently studied and characterized biopolymer PHA [2]. It is well-known for interesting thermoplastic properties, including resistance to a wide range of temperature from 30 to 120 °C. Furthermore, PHB is non-toxic biopolymer. When it degrades, it produces 3-hydroxybutyric acid, which can be found normally in human blood [9]. For these reasons, several companies, including Mitsubishi in Japan and Biomer in Germany, have produced PHB at both the pilot and research scales [10].

Biosynthesis of PHB requires PHA synthase, beta-ketothiolase, and reductase encoded by the *phaCAB* operon in many bacterial species, including *Ralstonia eutropha* [11] and *Pseudomonas putida* [4]. The model bacterial host, *Escherichia coli*, and some natural producers (mostly gram-negative bacterial strains) have been commonly used for the production of PHAs [2]. However, abundant endotoxins from gram-negative bacteria, including *E. coli* and PHA-producing *Ralstonia eutropha*, can provoke strong innate immune responses [12].

*Corynebacterium glutamicum* is a non-sporulating gram-positive bacterium with a ‘generally recognized as safe’ (GRAS) status that has been extensively employed for the industrial production of several food-grade amino acids and pharmaceutical products for decades [13, 14]. *C. glutamicum* has garnered increasing attention for its potential as a platform strain for industrial applications because of its unique features as a robust and GRAS microbial factory [13]. The product spectrum of *C. glutamicum* is expanding rapidly with the development of various genetic tools and strategies, including synthetic promoters [14, 15], signal peptides [16–18], transcription factor-based biosensors [19, 20], recombineering [21, 22], and clustered regularly interspaced short palindromic repeats (CRISPR) [22, 23].

*C. glutamicum* has also been employed for the production of PHB [24–26] and other PHA bioplastics [27] from various carbon substrates, including lignocellulosic biomass [26]. While overexpression of a heterologous PHA biosynthetic pathway by using strong promoters and codon optimization [24, 28] has been a widely employed strategy to improve PHA production levels in *C. glutamicum*, relocation of a PHA synthase into the cellular membrane and modification of the cell morphology have also been attempted [26].

Here, we describe a scalable and rapid strategy for rewiring global metabolic networks for the production of the PHB bioplastic in *C. glutamicum*. By staining intracellular PHB granules using BODIPY fluorescent dye, our framework enables fluorescence-activated cell sorting (FACS)-based high-throughput screening of a large combinatorial metabolic network library for enhanced production of PHB. We applied this approach to optimize the central carbon metabolism for PHB biosynthesis in *C. glutamicum* on two different sugar substrates, glucose and fructose, in minimal media. The metabolic networks optimized for each sugar resulted in the highest cellular PHB productivities in *C. glutamicum* using a sole carbon source.

## Results

### Engineering of *R. eutropha *PHB synthesis pathway in *C. glutamicum*

**Fig. 1:**
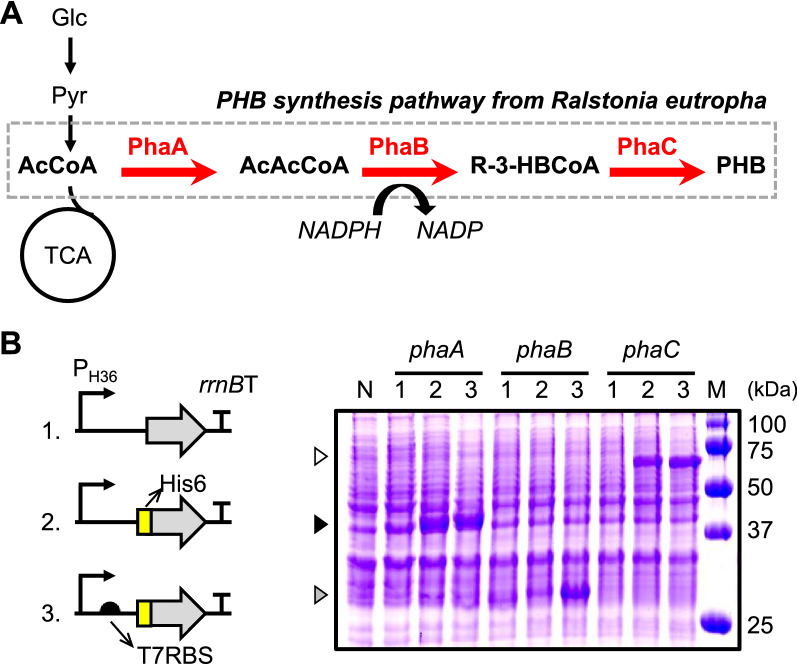
Engineering *R. eutropha* PHB synthesis pathway in *C. glutamicum*. **A** PHB biosynthetic metabolic pathway. The thick red arrows represent overexpression of genes in the PHB synthesis pathway. *Glc* glucose, *Pyr* pyruvate, *AcCoA* acetyl-CoA, *AcAcCoA* acetoacetyl-CoA, *R-3-HBCoA* R-3-hydroxybutyrl-CoA, *PHB* polyhydroxybutyrate, *TCA* tricarboxylic acid cycle. **B** Expression systems for *phaA, phaB, phaC* genes (left panel) and SDS-PAGE analysis of gene expression in *C. glutamicum* (right panel). *P*_*H36*_ H36 synthetic promoter, *rrnBT* rrnBT1T2 terminator, *H* N-terminal 6 × His tag, *T7RBS*T7 *gene10* RBS. Lane N, negative control *C. glutamicum* cells harboring pCES208 empty vector; Lanes 1 to 3, Expression systems 1 to 3; Lane M, Molecular weight markers. White, black and gray triangles indicate the bands of PhaC, PhaA and PhaB, respectively

To produce PHB in *C. glutamicum*, we first introduced the biosynthetic pathway for PHB in *R. eutropha*. The pathway consists of three genes (*phaA, phaB,* and *phaC*) and uses acetyl-CoA as a precursor and NADPH as a cofactor (Fig. 1A). To drive highly efficient gene expression, we placed a strong synthetic promoter, P_H36_, that was previously isolated from a fully synthetic promoter library constructed in *C. glutamicum* by our group [14]. However, overexpression was not observed for any of the target genes (Fig. 1B). We attempted to improve translational efficiency by modifying the N-terminal sequence of each gene with an additional 6 × His tag, which reportedly enhances the expression levels of many genes [29, 30]. The N-terminal 6 × His tag significantly improved the expression levels of *phaA* and *phaC*, but not *phaB* (Fig. 1B). We further added T7 *gene10* RBS, a well-known strong ribosomal binding site [29, 30], in the upstream of *phaA*, *phaC*, and *phaB* to induce efficient translation. The final combination of P_H36_, T7 *gene10* RBS, and the 6 × His tag drove the highly efficient gene expression of *phaA*, *phaB*, and *phaC* genes originated from *R. eutropha* in *C. glutamicum* (Fig. 1B).

**Fig. 2:**
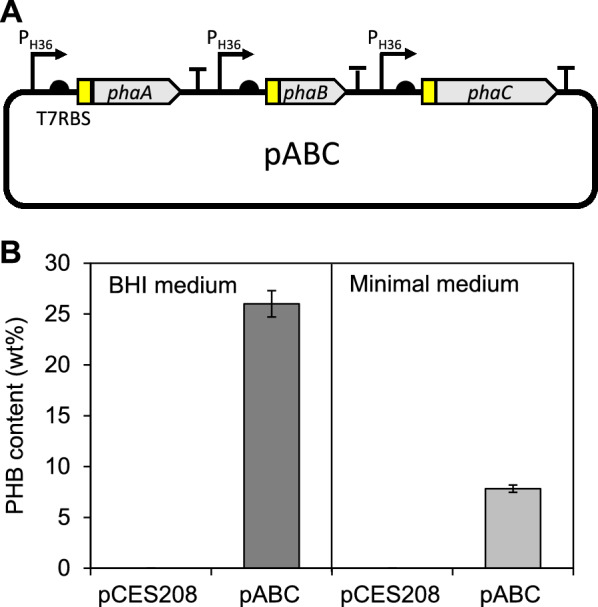
Construction of expression systems of PHB biosynthesis genes and PHB production** A** Schematic illustration of pABC for PHB biosynthesis genes expression in *C. glutamicum* **B** Comparison of PHB production levels of *C. glutamicum* harboring pABC in nutrient-rich (BHI) medium and in a minimal medium. Both media were supplemented with 2% glucose as a sole carbon source. *C. glutamicum* strain with an empty vector (pCES208) was used as a negative control. All measurements are based on two biological replicates. Error bars represent standard deviation of the biological replicates

Having established a highly efficient gene expression system for all three genes involved in PHB biosynthesis, we assembled the genes into a complete biosynthetic pathway using the type IIS restriction enzyme-based golden gate assembly technique [31] (Additional file 1: Fig. S1A). Each gene cassette, from promoter to gene and terminator, was amplified by polymerase chain reaction (PCR). The PCR products were cloned into pCES208-SapI to yield pABC containing the assembled PHB synthesis genes (Fig. 2A). *C. glutamicum* strain harboring pABC was cultivated in a minimal medium containing 2% glucose as a sole carbon source. The cellular PHB content was 7.3% of dry cell weight. In contrast, a control *C. glutamicum* strain with an empty pCES208 plasmid did not produce PHB (Fig. 2B). The findings indicate that the engineered PHB synthesis pathway in *R. eutropha* is functional in *C. glutamicum*. However, the production level achieved in a minimal medium was significantly lower than the level (~ 26% of dry cell weight) achieved in nutrient-rich brain heart infusion (BHI) medium, probably due to the limited supply of precursors and cofactors for PHB synthesis in a minimal medium culture conditions (Fig. 2B). The use of complex nutrient sources in culture media is one of the major technical hurdles to overcome in the development of economical fermentation routes [32]. To address the limited PHB productivity in a minimal medium, we sought to rewire the metabolic networks in *C. glutamicum* by screening a large number of metabolic variants.

### Rapid FACS-based quantification of cellular PHB contents

**Fig. 3:**
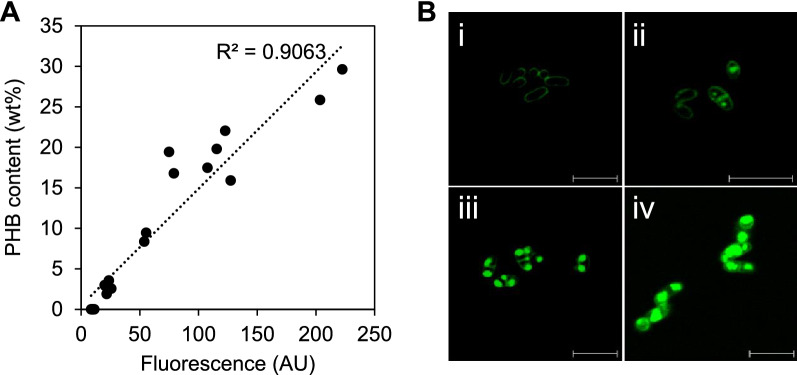
Fluorescence-based quantification assay for PHB content in *C. glutamicum*. **A** Intracellular PHB contents are linearly correlated with fluorescence intensities of BODIPY-stained PHB granules in cells. **B** Confocal microscope images of BODIPY-stained PHB-producing cells. (i) *C. glutamicum* harboring pCES208, 15 h cultivation in minimal media containing 2% glucose (PHB content: 0 wt%); (ii) *C. glutamicum* harboring pABC, 15 h cultivation in minimal media containing 2% glucose (PHB content: 3 wt%); (iii) *C. glutamicum* harboring pABC, 15 h cultivation in BHI media containing 2% glucose (PHB content: 12 wt%); iv, *C. glutamicum* harboring pABC, 36 h cultivation in BHI media containing 2% glucose (PHB content: 25 wt%)

There have been several attempts to screen a library of cells for increased PHB productivity. Most relied on the laborious characterization of intracellular PHB content using gas chromatography (GC) [33]. To expedite the quantification of PHB in single *C. glutamicum* cells in a large population of metabolic variants, we investigated the use of BODIPY, a lipophilic fluorescent dye, which has been widely used in labeling and estimating the intracellular content of lipids and polyhydroxyalkanoates in diverse microorganisms [34–37]. Compared to Nile Red dye, we previously found that BODIPY is more effective in staining intracellular PHB, more resistant to photobleaching, and with lower cellular toxicity in bacteria [34]. To generate cell populations with a wide range of cellular PHB contents, *C. glutamicum* harboring pABC was cultivated in minimal or BHI medium, and sampled at multiple time points. Intracellular PHB content in the sampled cell pellets was analyzed by conventional GC to determine the actual PHB content and by flow cytometry to determine the fluorescence intensity of BODIPY-stained PHB in cells. The actual PHB content and fluorescence intensity of BODIPY-stained PHB were linearly correlated with a coefficient of determination of 0.9063 (Fig. 3A). We further examined BODIPY-stained PHB in cells using confocal microscopy and confirmed the presence of BODIPY-stained intracellular PHB granules. These results demonstrate that BODIPY can be used to label cellular PHB in *C. glutamicum* to directly determine the PHB content through fluorescence intensity (Fig. 3B).

### Combinatorial optimization of metabolic networks for PHB production

**Fig. 4:**
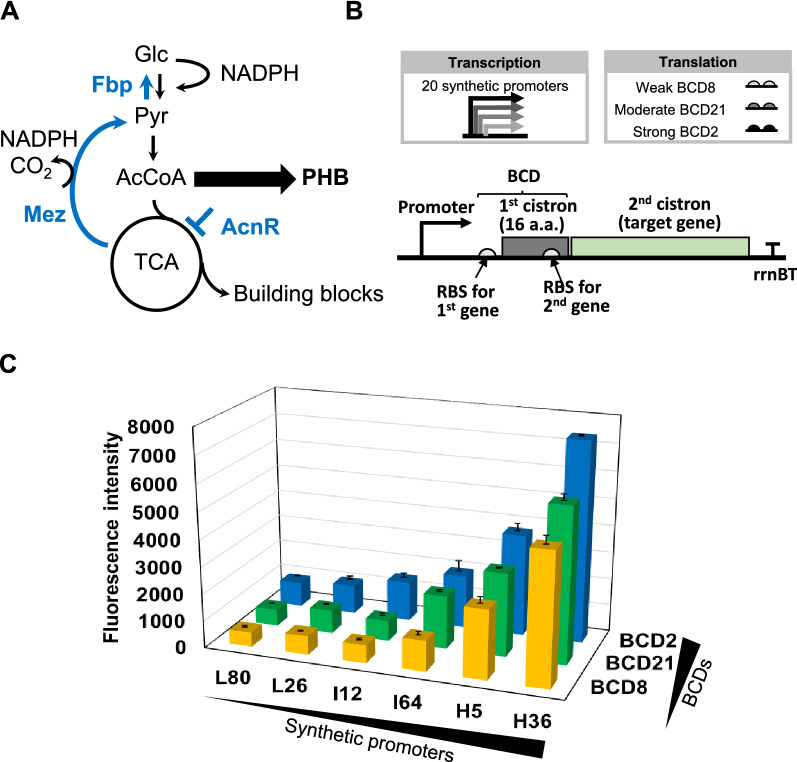
Metabolic network of PHB biosynthesis in *C. glutamicum* and gene expression in bicistronic design. **A** Metabolic network optimization for PHB synthesis by fine-tuning of gene expression levels of the *fbp*, *acnR*, *mez* genes. **B** Schematic illustration of bicistronic expression system with 20 synthetic promoters and bicistronic design of RBS (BCD) in *C. glutamicum*. **C** Comparison of GFP gene expression using six synthetic promoters and three BCDs in bicistronic expression system

Efficient biosynthesis cannot be achieved simply by overexpressing genes through a specific pathway. Metabolic imbalance and lack of precursor and cofactor pools significantly affect production yield [38]. PHB biosynthesis requires acetyl-CoA as a precursor and NADPH as a cofactor [2]. Acetyl-CoA and NADPH are also required for many other important metabolic activities for cell maintenance and growth [39], which makes them difficult to balance. To address this issue, we sought to rewire global metabolic networks for PHB production using a combinatorial metabolic engineering approach. To tune the NADPH level, carbon flux through the oxidative pentose phosphate pathway, which is the main source of NADPH generation in *C. glutamicum*, was targeted. We selected a gluconeogenic enzyme, fructose biphosphatase (Fbp) [40]. Fbp catalyzes the conversion of fructose 1,6-biphosphate to fructose 6-phosphate, and has been employed to redirect the carbon flux from glycogenesis into the pentose phosphate pathway (Fig. 4A) [41]. We also sought to tune carbon flux into the tricarboxylic acid (TCA) cycle and control the acetyl-CoA pool in cells [42]. For this purpose, a TetR-type transcription factor (AcnR) was selected as the second target. AcnR is a regulator of aconitase coding gene in *C. glutamicum* and can bind to a region between -35 and -10 motifs of *acn* promoter to downregulate the expression of aconitase gene within the TCA cycle [43]. The third target selected was NADP-dependent malate dehydrogenase (Mez) [44] which catalyzes the reversible oxidative decarboxylation of malate to pyruvate, CO_2_, and NADPH, regulating the reducing power and precursor pool simultaneously for PHB biosynthesis [45]. The genes encoding these three targets (*fbp, acnR,* and *mez*) were selected to affect across the whole central carbon metabolism in *C. glutamicum* (Fig. 4A).

To precisely control the expression levels of target genes, we designed 60 combinations of 20 synthetic promoters [14] for transcriptional control and three bicistronic designs [46] of RBS (BCD) for translational control (Fig. 4B). In the bicistronic design, the first cistron encodes a short peptide (16 amino acids) with its stop codon overlapped by one base pair with the start codon of the second cistron (target gene). Through translational coupling between the first and second cistrons, the expression of target genes can be controlled precisely and reliably by the strength of promoters and BCDs upstream of the target gene. To explore gene expression in the bicistronic design, we first tested 18 representative combinations of six synthetic promoters with different strengths (L80 < L26 < I12 < I64 < H5 < H36) and three BCDs, including weak BCD8, moderate BCD21, and strong BCD2, using green fluorescent protein (GFP) as a reporter. Each combination showed a much broader range of gene expression with narrower intervals compared to the range and interval that can be achieved by synthetic promoters alone. The findings demonstrating their utility in the precise and reliable control of gene expression (Fig. 4C).

A combinatorial metabolic network library of *fbp, acnR,* and *mez* was then assembled and inserted into the pABC plasmid containing the engineered PHB synthesis pathway (Additional file 1: Fig. S1B). The ligated library plasmids were introduced into *Escherichia coli*. More than 10^6^ *E. coli* transformants were obtained, which was sufficient to cover the theoretical diversity of the library (2.16 × 10^5^). *C. glutamicum* cells were transformed with the library and 5 × 10^5^ transformants were obtained. To confirm the library quality, colonies were randomly picked from the *C. glutamicum* library and sequence for their assembled constructs were determined. About 80% of the randomly selected clones were confirmed by Sanger sequencing to have all *fbp, acnR,* and *mez* genes with all different combinations of promoters and BCDs for each gene without any error (data not shown).

**Fig. 5:**
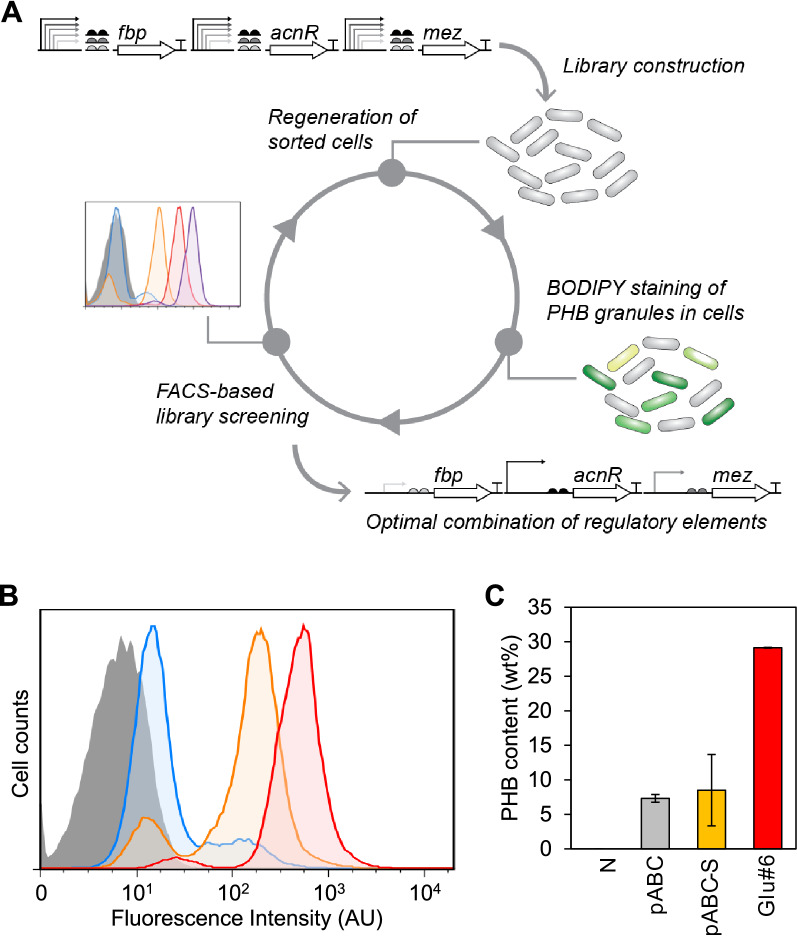
FACS-based high-throughput screening of metabolic network library for enhanced production of PHB **A** Schematic diagram of FACS-based screening strategy of the metabolic network library for enhanced production of PHB. **B** FACS-based screening of metabolic network library using glucose as a carbon substrate. The histograms of negative control (pCES208), original library, 1st sorted library, and 2nd sorted library cells are represented by filled-gray, tinted-blue, orange, and red lines, respectively. **C** PHB production levels using glucose as a sole carbon source in *C. glutamicum* strains harboring empty vector (pCES208), engineered PHB synthesis pathways (pABC and pABC-S), and the best clone isolated from metabolic network library (Glu#6). All measurements are based on two biological replicates. Error bars represent standard deviation of the biological replicates

Using this combinatorial library, we sought to isolate high PHB producers using a FACS-based high-throughput screening strategy (Fig. 5A). *C. glutamicum* library cells were cultivated on glucose in a minimal medium for 24 h and stained with BODIPY. The PHB content of the individual cells in the library population was examined using flow cytometry. As shown in Fig. 5B, the distribution of fluorescence intensities from individual cells was higher in the library cell population than that in the negative control cell population lacking the PHB synthesis pathway. We collected the top 1% of fluorescent cells in the library, and the cells were grown overnight for subsequent rounds of screening. During the two rounds of FACS-based screening, highly fluorescent cells were enriched in the library population (Fig. 5B), and no further enrichment was observed with more iterations.

After the second round of sorting, 10 variants were randomly selected from the enriched population. Their promoter-BCD combinations for each targeted gene and PHB content were examined. All the clones had different combinations of promoter and BCD, and most had significantly higher PHB content than the pABC-harboring cells (Additional file 1: Fig. S2A). The Glu#6 variant displayed the highest PHB content (29.1% of dry cell weight) from 2% glucose in a minimal medium. This variant contains the I64 promoter with strong BCD2 for *fbp* expression, I12 promoter with moderate BCD21 for *acnR* expression, and H36 promoter with weak BCD8 for *mez* expression. This PHB production yield was fourfold higher than that of *C. glutamicum* cells with the pABC plasmid without metabolic network balancing and slightly higher than that of *C. glutamicum* cells grown in nutrient-rich complex BHI medium (Fig. 5C). In addition, we examined the PHB production yield of *C. glutamicum* cells harboring the pABC-S plasmid that contained the strongest regulatory elements (H36 synthetic promoter and BCD2) for all three target genes (*fbp*, *acnR*, and *mez*). Interestingly, *C. glutamicum* with pABC-S showed a marginal increase in PHB content (8.5% of dry cell weight) (Fig. 5C), highlighting the importance of precise metabolic rebalancing for optimal product formation.

### Isolation of high PHB producer using fructose as a sole carbon source

**Fig. 6:**
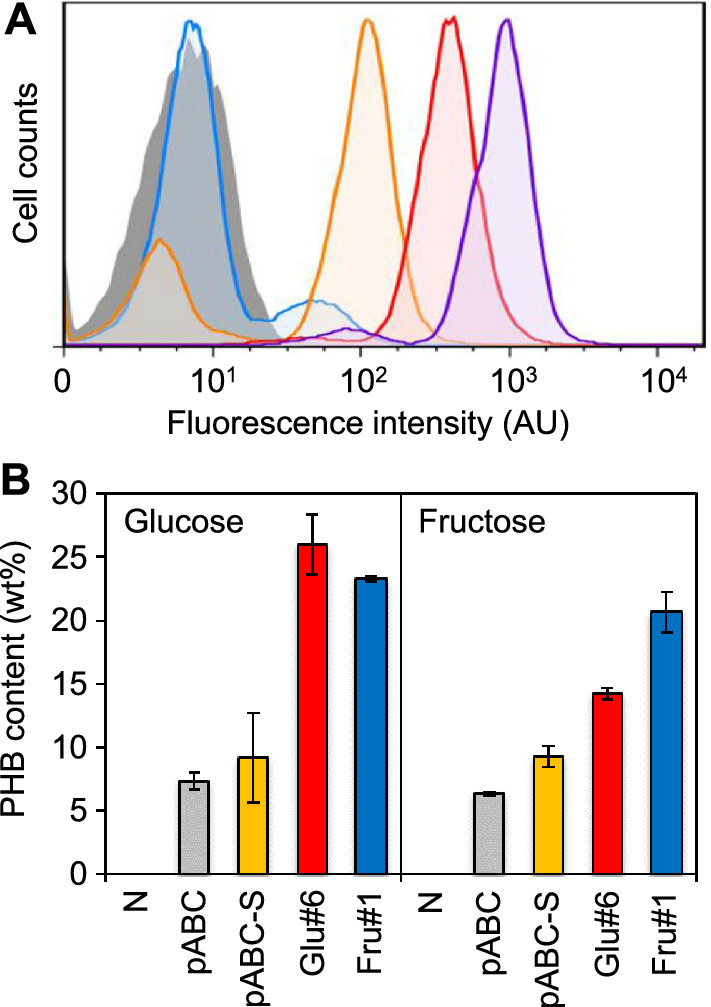
Engineering carbon substrate-specific metabolic networks in *C. glutamicum* for PHB production. **A** FACS-based screening of the metabolic network library using fructose as a carbon substrate. The histograms of negative control (pCES208), original library, 1^st^ sorted library, 2^nd^ sorted library, and 3^rd^ sorted library cells are represented by filled-gray, tinted-blue, orange, red, and purple lines, respectively. **B** PHB production levels using glucose or fructose as a sole carbon source in *C. glutamicum* strains harboring empty vector (pCES208), engineered PHB synthesis pathways (pABC and pABC-S), and the isolated clones from the metabolic network library (Glu#6 and Fru#1). All measurements are based on two biological replicates. Error bars represent standard deviation of the biological replicates

To further demonstrate the utility of our strategy to rapidly optimize metabolic networks on different carbon substrates, we screened the metabolic network library of *C. glutamicum* again using fructose as a sole carbon source in a minimal medium. After three rounds of FACS-based library screening, the highly fluorescent cell population was enriched (Fig. 6A). Since fructose enters glycolysis in the form of fructose-1,6-biphosphate, resulting in low carbon flux through the pentose phosphate pathway, we expected that the variants in the enriched population would have stronger regulatory elements for *fbp* and weaker regulatory elements for *acnR* than Glu#6 towards increased NADPH supply. Indeed, five randomly selected colonies were identical (named Fru#1). Fru#1 contained the H17 promoter with weak BCD8 for *fbp*, L10 promoter with moderate BCD21 for *acnR*, and H30 promoter with weak BCD8 for *mez*. To assess whether their metabolic network was specifically optimized for fructose, we compared the cellular PHB content of Fru#1 and Glu#6 grown on fructose or glucose as a sole carbon source in a minimal medium. Interestingly, Glu#6 showed a higher PHB production yield than Fru#1 on glucose, and Fru#1 showed a higher yield than Glu#6 on fructose (Fig. 6B). Together, these results demonstrate that the global metabolic network can be rewired specifically for certain carbon substrates to enhance the production of target biochemicals.

## Discussion

According to Market Research Future (www.marketresearchfuture.com), the PHB market was valued at ~ 62 million US dollars in 2018, and it is expected to grow to ~ 121 million US dollars by the end of 2028 in the global market. While the cost of manufacturing PHB is 20–80% higher than conventional plastics, increasing demand for sustainable and biodegradable bioplastics, especially for biomedical applications, is fueling the market growth for PHB. To address this growing market demand for biomedical bioplastics, *C. glutamicum* has been utilized as a potential host for the production of diverse PHA bioplastics, including PHB, poly(3-hydroxybutyrate-co-3-hydroxyvalerate) (PHBV), and poly(lactate-co-3-hydroxy butyrate) (PLAHB) (Table 1). In most cases, heterologous genes in PHA biosynthetic pathways (mostly from *R. eutropha*) were overexpressed (on) and some genes in competing metabolic pathways were removed (off) in *C. glutamicum*. As such, gene expression has been simply turned on or off to control PHB production in the previous studies. In the present work, we reported a FACS-based fine-rewiring of metabolic network for the first time to improve PHA production. We established a fluorescence-based quantification assay for cellular PHB content using BODIPY in *C. glutamicum* to screen a large combinatorial metabolic library of *C. glutamicum* cells. To demonstrate the utility of our approach, glucose and fructose were selected as a sole starting carbon source for the following reasons. First, from a practical standpoint, *C. glutamicum* can naturally metabolize glucose and fructose as a carbon source without introduction of heterologous genes. Second, glucose and fructose are well known to affect central carbon metabolism differently in *C. glutamicum*. Although the entry points of the sugars are not so far apart, 60% of carbon flux goes into oxidative pentose phosphate pathway (PPP) on glucose and only 10% goes into PPP on fructose [47]. Consequently, the biosynthesis of NADPH demanding PHB could be affected significantly when the cells are grown on fructose. Indeed, different combinations of regulatory elements for *fbp, acnR,* and *mez* genes were found to be optimal for PHB production on glucose and fructose in our study. Especially, *fbp* expression, which can redirect carbon flux into PPP, was highly upregulated in the fructose-optimized metabolic network than in the glucose-optimized one (H17 ‘high’ promoter in Fru#1, I64 ‘intermediate’ promoter in Glu#6) [14, 48].

**Table 1:** Summary of polyhydroxyalkanoates (PHAs) production in *C. glutamicum*

PHAs	Source of the biosynthetic genes	Carbon source	Max. PHA content (wt%)	Cellular productivity^a^	Refs.
PHB, poly(3-hydroxybutyrate)	*R. eutropha*	20 g/L glucose in LB media	12.1	0.0252	[25]
	*R. eutropha*	60 g/L glucose in minimal media	22.5	0.0078	[24]
	*R. eutropha*	60 g/L glucose in minimal media	52.5	0.0156	[28]
	*R. eutropha*	20 g /L glucose in minimal media	29.1	0.0404	This study
	*R. eutropha*	20 g/L fructose in minimal media	21	0.0292	This study
PHBV, poly(3-hydroxybutyrate-co-3-hydroxyvalerate)	*R. eutropha*	130 g/L glucose in minimal media	28.7	0.0023	[27]
	*R. eutropha*	60 g/L glucose in minimal media	47.2	0.0187	[54]
PLAHB, poly(lactate-co-3-hydroxybutyrate)	*Pseudomonas* sp., *R. eturopha,* *Megasphaera elsdenii*	60 g/L glucose in minimal media	2.4	0.0006	[12]

^a^PHB content per g carbon source per liter per hour

There have been many reports of PHB production in model industrial bacteria, *Escherichia coli,* and some of natural PHB producers, including *Pseudomonas putida* and *Ralstonia eutropha,* since its first discovery as a lipid-like inclusion body in *Bacillus megaterium* in 1926 [2]. While PHB production levels vary across microbial hosts and their culture conditions (including carbon sources and culture modes), their cellular PHB contents could even reach above 80 wt% [2] and are much higher than the maximum content (29.1 wt%) achieved in this study. However, abundant endotoxins from gram-negative bacteria, including *E. coli* and PHA-producing *R. eutropha,* can provoke strong innate immune responses. In addition, most endotoxin-free Gram-positive PHB producers such as *B. megaterium*, produce spores and therefore are not suitable for industrial applications without extensive genetic engineering. On the other hand, *C. glutamicum* is a ‘non-sporulating’ gram-positive ‘industrial’ bacterium with a ‘generally recognized as safe’ (GRAS) status. These unique characteristics make *C. glutamicum* as an attractive, robust, and safe platform to produce bioplastics for biomedical applications in industrial scales.

To the best of our knowledge, optimization of culture conditions has always been conducted after all strain engineering efforts for the industrial scale production of biochemicals. However, our results demonstrate that it is critical to engineer producer strains based on the specific carbon source, medium, and culture conditions that will be employed in industrially relevant production conditions. The use of other carbon sources could be either beneficial or detrimental based on their effects on central carbon metabolism and intracellular pool of the precursors and cofactors. While we could screen for alternative carbon sources that boost up the cellular productivity of PHB, we sought to take control on cellular metabolism to optimize metabolic fluxes toward highest possible PHB production for any carbon source in *C. glutamicum* in this study. We believe that our approach could potentially be applied to various biochemicals in diverse microbial or eukaryotic hosts on many different single or mixed carbon sources. For example, BODIPY have been used in staining of PHAs and other lipid-related granules in microbial and microalgal hosts [34, 36], so we expect that a similar metabolic rewiring approach could easily be employed in host engineering to enhance the production of lipid biofuels. We also expect our high-throughput FACS-based approach could be employed to evolve a PHA-producing microbial population for higher productivities [49]. In addition, one could use transcription factors, riboswitches, or biosensor systems based on Förster resonance energy transfer to further expand the target biochemical spectrum beyond lipids and PHAs [20, 50, 51].

## Conclusions

In this study, we successfully engineered a heterologous PHB biosynthetic pathway derived from *R. eutropha* and rapidly optimized metabolic networks across central metabolism in *C. glutamicum* for enhanced production of PHB on a sole carbon sources in a minimal medium. Through FACS-based combinatorial optimization of *fbp, acnR,* and *mez* gene expression, outperforming *C. glutamicum* variants could be isolated. On glucose, the isolated variant showed ~ 3.5-fold higher PHB content (29.1% of dry cell weight) compared to non-optimal strain (8.5% of dry cell weight) with all strong combinations of regulatory elements for the target genes. Furthermore, we successfully demonstrated that, for efficient product formation, metabolic networks need to be optimized for each of different carbon substrates. We believe that this study provides a foundation for accelerating the design-build-test-learn (D-B-T-L) cycle for strain engineering in the industrial production of various biochemicals.

## Materials and methods

### Bacterial strains and growth conditions

The bacterial strains and plasmids used in this study are listed in Table 2. *E. coli* XL1-Blue was used as the host for gene cloning and plasmid maintenance, and *C. glutamicum* ATCC 13032 was used as the main host for gene expression and PHB production. For gene expression in *C. glutamicum* strains, the plasmids pCES208, *E. coli-C. glutamicum* shuttle vector was used as the main plasmid [34]. PCR was performed using a C1000™ Thermal Cycler (Bio-Rad, Hercules, CA, USA) with PrimeSTAR HS polymerase (TaKaRa Bio Inc., Shiga, Japan). The nucleotide sequences of all the primers used in this study are listed in Additional file 2: Table S1.

**Table 2:** Bacterial strains and plasmids used in this study

Strain	Relevant characteristics	Refs. or Source
XL1-Blue	*recA1 endA1 gyrA96 thi-1 hsdR17 supE44 relA1 lac [F´ proAB lacIqZ∆M15 Tn10 (Tet*^*r*^*)]*	Stratagene^a^
*C. glutamicum*	Wild type	ATCC 13032
Plasmids	Relevant Characteristics	Ref. or Source
pCES208	*E. coli-C. glutamicum* shuttle vector; Km^R^	[43]
pCG-H36A	pCES208 derivative; P_H36_, Signal sequence of *cg1514*	[14]
pCES-H36-GFP	pCES208 derivative; P_H36_, GFP	[12]
pCES208-SapI	pCES208 derivative; two additional *Sap*I restriction enzyme sites	This study
pA	pCES208 derivative; P_H36_, *phaA* from *R. eutropha*, *rrnB* T1T2 terminator	This study
pHA	pCES208 derivative; P_H36_, *phaA* from *R. eutropha* with N-terminal 6 × His tag, *rrnB* T1T2	This study
pUHA	pCES208 derivative; P_H36_, T7 *g10* RBS, *phaA* from *R. eutropha* with N-terminal 6 × His tag, *rrnB* T1T2	This study
pB	pCES208 derivative; P_H36_, *phaB* from *R. eutropha*, *rrnB* T1T2 terminator	This study
pHB	pCES208 derivative; P_H36_, *phaB* from *R. eutropha* with N-terminal 6 × His tag, *rrnB* T1T2 terminator	This study
pUHB	pCES208 derivative; P_H36_, T7 *g10* RBS, *phaB* from *R. eutropha* with N-terminal 6 × His tag, *rrnB* T1T2	This study
pC	pCES208 derivative; P_H36_, *phaC* from *R. eutropha*, *rrnB* T1T2 terminator	This study
pHC	pCES208 derivative; P_H36_, *phaC* from *R. eutropha* with N-terminal 6 × His tag, *rrnB* T1T2 terminator	This study
pUHC	pCES208 derivative; P_H36_, T7 *g10* RBS, *phaC* from *R. eutropha* with N-terminal 6 × His tag, *rrnB* T1T2	This study
pABC	pCES208 derivative; P_H36_-T7 *g10* RBS-*phaA* with N-terminal 6 × His tag-*rrnB* T1T2; P_H36_-T7 *g10* RBS-*phaB* with N-terminal 6 × His tag-*rrnB* T1T2; P_H36_-T7 *g10* RBS-*phaC* with N-terminal 6 × His tag-*rrnB* T1T2	This study
pABC-S	pABC derivative; P_H36_-BCD2-*fbp*-*rrnB*T1T2; P_H36_-BCD2-*acnR*-*rrnB*T1T2; P_H36_-BCD2-*mez*-*rrnB*T1T2	This study
Glu#6	pCES208 derivative; P_I64_-BCD21-*fbp*-*rrnB*T1T2; P_I64_-BCD21-*acnR*-*rrnB*T1T2; P_H36_-BCD8-*mez*-*rnB*T1T2	This study
Fru#1	pCES208 derivative; P_H17_-BCD8-*fbp*-*rrnB*T1T2; P_L10_-BCD21-*acnR*-*rrnB*T1T2; P_H30_-BCD8-*mez-rrnB*T1T2	This study

^a^Stratagene, La Jolla, CA, USA

For plasmid preparation, *E. coli* was cultivated in Luria–Bertani broth (tryptone, 10 g/L; yeast extract, 5 g/L; and NaCl, 10 g/L) at 37 °C. For cultivation of *C. glutamicum* strains, BHI (Difco Laboratories, Detroit, MI, USA) and a minimal medium (3 g/L K_2_HPO4, 1 g/L KH_2_PO_4_, 2 g/L urea, 10 g/L (NH_4_)_2_SO_4_, 2 g/L MgSO_4_, 200 μg/L biotin, 5 g/L thiamine, 10 g/L MnSO_4_, 1 g/L ZnSO_4_, and 10 mg/L CaCl_2_) were used with 20 g/L sugar carbon substrate. *C. glutamicum* cells were inoculated into BHI medium and grown at 30 °C for 24 h. Aliquots (500 μL) were transferred into 100 mL minimal media in 250-mL Erlenmeyer flasks and grown at 30 °C for 36 h. Kanamycin (Km, 25 μg/L) was added to the culture medium as a sole antibiotic.

### Plasmid manipulation and library construction

A graphical summary of plasmid and library construction is provided in Additional file 1: Fig. S1. For the expression of three PHB biosynthesis genes (*phaA*, *phaB*, and *phaC*) from *R. eutropha*, each gene was amplified from the chromosome of *R. eutropha* by PCR using primer pairs PhaA-F and PhaA-R, PhaB-F and PhaB-R, and PhaC-F and PhaC-R, respectively. After digestion with *Bam*HI and *Not*I, the PCR products (PhaA, PhaB, and PhaC) were cloned into pCG-H36A [16] between the strong H36 synthetic promoter and *rrnB*T1T2 terminator to yield pA, pB, and pC, respectively. To add a 6 × His tag (HHHHHH) to the N-terminus of PhaA, PhaB, and PhaC, each gene was amplified from the *R. eutropha* chromosome by PCR using primer pairs H-PhaA-F and PhaA-R, H-PhaB-F and PhaB-R, and H-PhaC-F and PhaC-R, respectively. After digestion with *Bam*HI and *Not*I, the PCR products were cloned into pCG-H36A [16] to yield pHA, pHB, and pHC, respectively. To introduce the T7 *gene10* RBS sequence to His-tagged *phaA, phaB,* and *phaC*, each gene was PCR amplified from the *R. eutropha* chromosome with the primer pairs UH-PhaA-F and PhaA-R, UH-PhaB-F and PhaB-R, and UH-PhaC-F and PhaC-R, respectively. After digestion with *Bam*HI and *Not*I, the PCR products were cloned into pCG-H36A [16] to yield pUHA, pUHB, and pUHC, respectively.

The pCES208-SapI plasmid is a derivative of the pCES208 shuttle vector with two additional *Sap*I-Type IIS restriction enzyme sites. For the introduction of *Sap*I restriction enzyme sites, a PCR fragment was amplified by PCR using the primer pair SapI-F and SapI-R. After digestion with *Nco*I, the PCR product was cloned into pCES208 to obtain pCES208-SapI. For construction of whole PHB synthesis pathway by combining the expression systems for *phaA, phaB,* and *phaC* from *R. eutropha*, which are all composed of H36 synthetic promoter, T7 *gene10* RBS, N-terminal 6 × His Tag, and *rrnB*T1T2 terminator, each expression system was amplified from pUHA, pUHB, and pUHC by PCR with primer pairs, A-BsaI-F and A-BsaI-R, B-BsaI-F and B-BsaI-R, and C-BsaI-F and C-BsaI-R, respectively. After digestion with *Bsa*I-type IIS restriction enzyme, all PCR products were combined with *Sal*I and NotI-treated pCES208-SapI for the assembly of expression systems for *phaA*, *phaB*, and *phaC* to yield pABC.

For the expression of the fructose biphosphatase (*fbp*), TetR-type aconitase repressor (*acnR*), and malate dehydrogenase (*mez*) genes from *C. glutamicum*, each gene was amplified using three different bicistronic design (BCD) of RBSs [46] from the *C. glutamicum* chromosome by PCR with primer pairs: BCD2-Fbp-F and Fbp-R, BCD21-Fbp-F and Fbp-R, BCD8-Fbp-F and Fbp-R, BCD2-AcnR-F and AcnR-R, BCD21-AcnR-F and AcnR-R, BCD8-AcnR-F and AcnR-R, BCD2-Mez-F and Mez-R, BCD21-Mez-F and Mez-R, and BCD8-Mez-F and Mez-R, respectively. In all reverse primers (Fbp-R, AcnR-R, and Mez-R), the *lpp* terminator sequence was used to add the sequence at the end of each gene. After digestion with *Bam*HI and *Not*I, the PCR products were cloned into pCES-H36-GFP [14] which has a strong H36 promoter for the expression of target genes to yield pCES-H36-BCD2-Fbp-T, pCES-H36-BCD21-Fbp-T, pCES-H36-BCD8-Fbp-T, pCES-H36-BCD2-AcnR-T, pCES-H36-BCD21-AcnR-T, pCES-H36-BCD8-AcnR-T, pCES-H36-BCD2-Mez-T, pCES-H36-BCD21-Mez-T, and pCES-H36-BCD8-Mez-T. To introduce 20 synthetic promoters (L10, L26, L80, I9, I12, I15, I16, I29, I51, I64, H3, H4, H5, H17, H28, H30, H34, H36, H43, and H72) previously developed by FACS-based screening of promoter library [14], each promoter was prepared from each clone by *Kpn*I and *Bam*HI digestion. Each fragment was cloned into pCES-H36-BCD2-Fbp-T, pCES-H36-BCD21-Fbp-T, pCES-H36-BCD8-Fbp-T, pCES-H36-BCD2-AcnR-T, pCES-H36-BCD21-AcnR-T, pCES-H36-BCD8-AcnR-T, pCES-H36-BCD2-Mez-T, pCES-H36-BCD21-Mez-T, and pCES-H36-BCD8-Mez-T to yield 60 combinations of promoters and BCDs for the expression of *fbp*, *acnR*, and *mez* genes, respectively. To construct a metabolic network library by combinatorial assembly of the expression systems for *fbp*, *acnR*, and *mez* genes, each pool of 60 combinations for each gene was PCR amplified with the primer pairs Fbp-SapI-F and Fbp-SapI-R, AcnR-SapI-F and AcnR-SapI-R, and Mez-SapI-F and Mez-SapI-R, respectively. After digestion with *Sap*I-Type IIS restriction enzyme, all digested PCR products were assembled into *Sap*I-treated pABC to yield a metabolic network library for PHB production. To construct a control plasmid with the strongest expression systems for all the *fbp*, *acnR*, and *mez* genes from *C. glutamicum*, in addition to the *phaA*, *phaB*, and *phaC* genes from *R. eutropha*, expression systems for *fbp*, *acnR*, and *mez* genes, which consist of the H36 synthetic promoter (strongest promoter) and BCD2 (strongest BCD), were PCR amplified with primer pairs Fbp-SapI-F and Fbp-SapI-R, AcnR-SapI-F and AcnR-SapI-R, and Mez-SapI-F and Mez-SapI-R, respectively. After digestion with *Sap*I-Type IIS restriction enzyme, all PCR products were combined with *Sap*I-treated pABC to yield pABC-S.

### Protein preparation and analysis

After cultivation, cells were harvested by centrifugation at 6000 rpm for 10 min at 4 °C. The cells were then washed twice with phosphate-buffered saline PBS (135 mM NaCl, 2.7 mM KCl, 4.3 mM Na_2_PO_4_, 1.4 mM KH_2_PO_4_, pH 7.2) and resuspended in the same buffer. Total cell lysates were prepared by sonication (7 min at 40% pulse and 20 amplitude) and the extracts were centrifuged at 10,000 rpm for 10 min at 4 °C to yield soluble lysates. The protein samples were stored at − 20 °C until further analysis. All protein samples were analyzed by 12% (w/v) SDS-PAGE. In SDS-PAGE analysis, samples were loaded on 12% polyacrylamide gels. After the gel electrophoresis, the gels were stained with Coomassie brilliant blue [50% (*v/v*) methanol, 10% (*v/v*) acetic acid, 1 g/L Coomassie brilliant blue R-250] for 1 h and destained using a destaining solution [10% (*v/v*) methanol, 10% (*v/v*) acetic acid] [52].

### GC-based quantification of cellular PHB content

Cellular PHB content was determined by gas chromatography using the 6890 N GC System (Agilent Technologies, Palo Alto, CA, USA) equipped with a fused silica capillary column (SPBTM-5, 30 m × 0.32 mm ID, 0.25 μm film; Supelco, Bellefonte, PA, USA) and benzoic acid as an internal standard [53]. Cell concentration, defined as the dry cell weight per liter of culture broth, was determined as previously described [34]. The PHB content (wt%) was defined as the ratio of PHB concentration to cell concentration.

### FACS-based assay for cellular PHB content

After cultivation, the cells were harvested by centrifugation at 6000 rpm for 10 min at 4 °C. Cells were washed twice with PBS and resuspended in the same buffer. Next, 5 μL of BODIPY solution dissolved in dimethyl sulfoxide at a concentration of 1 g/L was added and vigorously mixed with the cells by vortexing for 10 s. After 5 min of incubation on ice, cells were washed twice with PBS and resuspended in the same buffer. After staining, the cells were analyzed or sorted on a flow cytometer (MoFlo XDP, Beckman Coulter, Inc., Miami, FL, USA) based on fluorescence intensity through a 530/40 band-pass filter for the BODIPY emission spectrum. For the FACS sorting mode, a purification mode that can sort drops containing only positive cells was used. The sorted cells were recovered on solid BHI agar plates. Fully grown colonies were collected by scraping and grown in minimal medium for the next round of screening. All procedures and sorting procedures were repeated as described above until no further significant enrichment was observed.

### Confocal microscopy

After BODIPY staining, the cells were mounted on poly L-lysine-coated microscopic glass slides and examined using confocal microscopy (Carl Zeiss, Jena, Germany). Images were taken using a model LSM410 microscope (Carl Zeiss). The samples were excited at 488 nm and the images were filtered using a long-pass 505-nm filter.

## Supplementary Information

**Additional file 1: Figure S1.** Golden Gate assembly strategy to assemble pABC and metabolic network library.** A** Construction of PHB synthesis pathway. **B** Assembly of metabolic network library in *C. glutamicum*. Restriction enzyme sites are indicated by colored box (Red B: *Bsa*I, Red S: *Sal*I, Red N: *Not*I, Blue S: *Sap*I). **Figure S2. **Individual clones that were isolated from the metabolic network library using glucose as a carbon substrate. **A** PHB content of the isolated clones from the metabolic network library (Glu#1, 2, 4, 5, 6, 7, 8, 10) and the strains with engineered PHB synthesis pathways (pABC, pABC-S) or empty vector (pCES208, ‘N’). All measurements are based on two biological replicates. Error bars represent standard deviation of the biological replicates. **B** Promoter and BCD combinations for each of *fbp, acnR, mez* genes in the isolated clones.

**Additional file 2: Table S1.** List of oligonucleotides used in this study

## Data Availability

All data are available upon request.
