# Supplementary material for: Rapid combinatorial rewiring of metabolic networks for enhanced poly(3-hydroxybutyrate) production in Corynebacterium glutamicum
Source: Microb Cell Fact. 2023 Feb 17;22:29. doi: 10.1186/s12934-023-02037-x (PMC9936768; doi:10.1186/s12934-023-02037-x)
Supplement: Supplementary file 1 — Additional file 1: Figure S1. Golden Gate assembly strategy to assemble pABC and metabolic network library. A Construction of PHB synthesis pathway. B Assembly of metabolic network library in C. glutamicum. Restriction enzyme sites are indicated by colored box (Red B: BsaI, Red S: SalI, Red N: NotI, Blue S: SapI). Figure S2. Individual clones that were isolated from the metabolic network library using glucose as a carbon substrate. A PHB content of the isolated clones from the metabolic network library (Glu#1, 2, 4, 5, 6, 7, 8, 10) and the strains with engineered PHB synthesis pathways (pABC, pABC-S) or empty vector (pCES208, ‘N’). All measurements are based on two biological replicates. Error bars represent standard deviation of the biological replicates. B Promoter and BCD combinations for each of fbp, acnR, mez genes in the isolated clones. [file 12934_2023_2037_MOESM1_ESM.pdf]

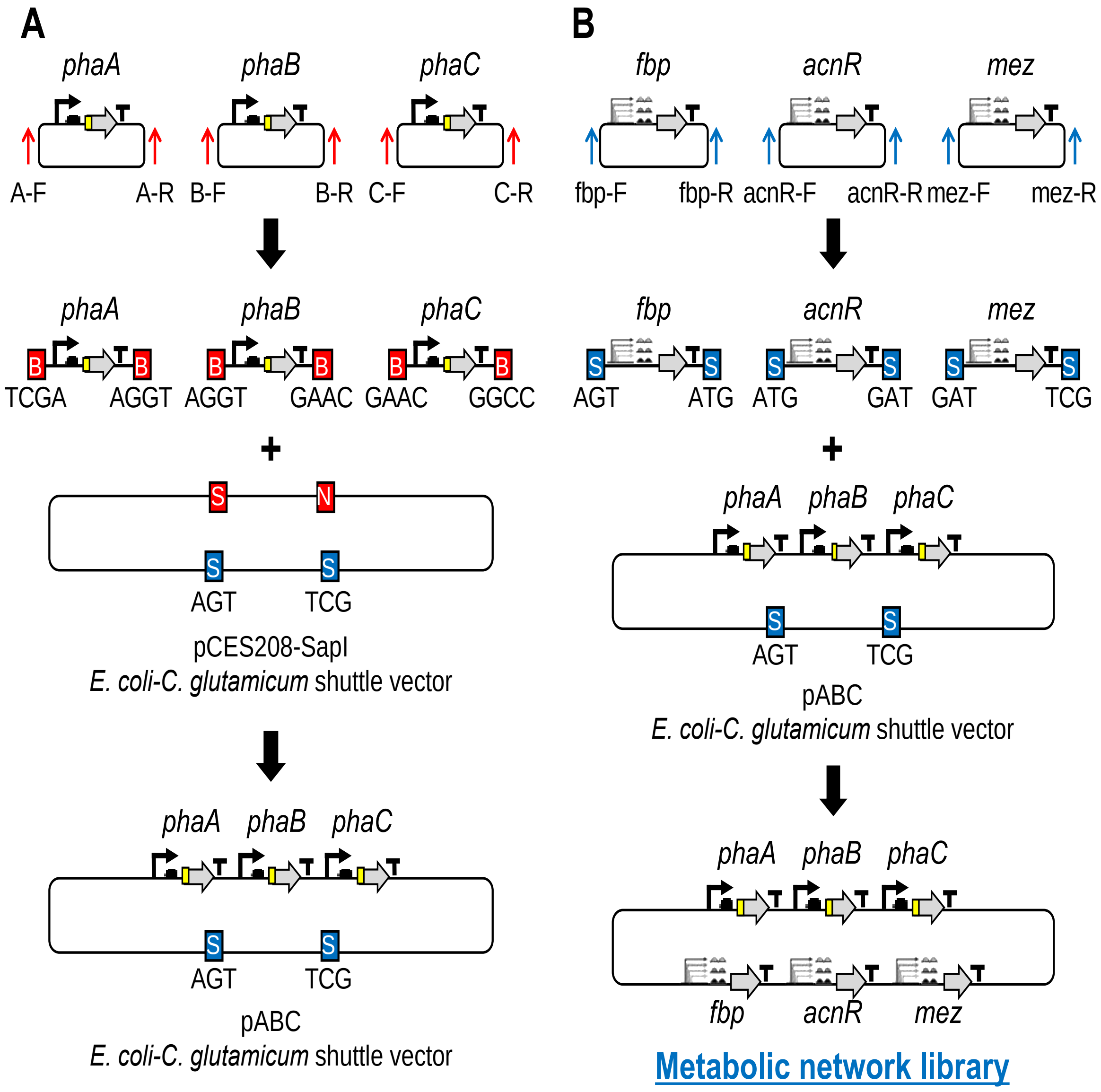

Figure S1. Golden Gate assembly strategy to assemble pABC and metabolic network library. (A) Construction of PHB synthesis pathway. **(B)** Assembly of metabolic network library in *C. glutamicum*. Restrction enzyme sites are indicated by colored box (Red B: *Bsa*I, Red S: *Sal*I, Red N: *Not*I, Blue S: *Sap*I).

A

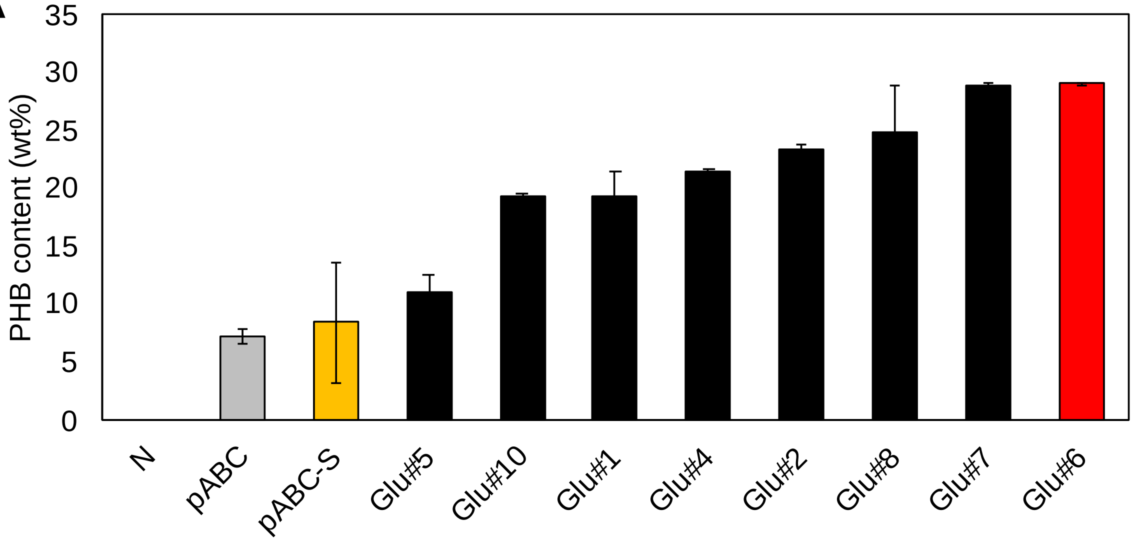

B

	Fbp		AcnR		Mez	
	Promoter	BCD	Promoter	BCD	Promoter	BCD
Glu#1	H3	BCD8	L10	BCD2	L10	BCD2
Glu#2	H28	BCD2	L10	BCD8	I64	BCD8
Glu#4	H4	BCD2	I15	BCD21	I15	BCD8
Glu#5	H28	BCD2	L10	BCD8	L10	BCD2
Glu#6	I64	BCD21	I12	BCD21	H36	BCD8
Glu#7	H17	BCD2	H28	BCD2	I12	BCD2
Glu#8	I12	BCD2	L26	BCD2	I64	BCD2
Glu#10	H17	BCD21	L10	BCD8	L10	BCD2

Figure S2. Individual clones that were isolated from the metabolic network library using glucose as a carbon substrate. (A) PHB content of the isolated clones from the metabolic network library (Glu#1, 2, 4, 5, 6, 7, 8, 10), strains with engineered PHB synthesis pathways (pABC, pABC-S), and empty vector (pCES208, 'N'). All measurements are based on two biological replicates. Error bars represent standard deviation of the biological replicates. **(B)** Promoter and BCD combinations for each of *fbp, acnR, mez* genes in the isolated clones.
