# Supplementary material for: Rapid combinatorial rewiring of metabolic networks for enhanced poly(3-hydroxybutyrate) production in Corynebacterium glutamicum
Source: Microb Cell Fact. 2023 Feb 17;22:29. doi: 10.1186/s12934-023-02037-x (PMC9936768; doi:10.1186/s12934-023-02037-x)
Supplement: Supplementary file 2 — Additional file 2: Table S1. List of oligonucleotides used in this study [file 12934_2023_2037_MOESM2_ESM.pdf]

Table S1. List of oligonucleotides used in this study

Primers	Sequence
PhaA-F	ATTAATGGATCCATGACTGACGTTGTCATCGTATC
PhaA-R	ATTAATGCGGCCGCTTACTATTTGTCATCGTCATCTTTATAATCTTTGC GCTCGACTGC
PhaB-F	ATTAATGGATCCATGACTCAGCGCATTGCGTAT
PhaB-R	ATTAATGCGGCCGCTTACTATTTGTCATCGTCATCTTTATAATCGCCCA TGTGCAGGC
PhaC-F	ATTAATGGATCCATGGCGACCGGCAAAGGC
PhaC-R	ATTAATGCGGCCGCTTACTATTTGTCATCGTCATCTTTATAATCTGCCTT GGCTTTGACGTATC
H-PhaA-F	ATTAATGGATCCATGCATCACCATCACCATCATACTGACGTTGTCATCG TATC
H-PhaB-F	ATTAATGGATCCATGCATCACCATCACCATCATACTCAGCGCATTGCGT AT
H-PhaC-F	ATTAATGGATCCATGCATCACCATCACCATCATGCGACCGGCAAAGGC
UH-PhaA-F	ATTAATGGATCCTCTAGATAACTTTAAGAAGGAGATATACATATGACT GACGTTGTCATCGTATC
UH-PhaB-F	ATTAATGGATCCTCTAGATAACTTTAAGAAGGAGATATACATATGACT CAGCGCATTGCGTAT
UH-PhaC-F	ATTAATGGATCCTCTAGATAACTTTAAGAAGGAGATATACATATGGCG ACCGGCAAAGGC
SapI-F	ATTAATCCATGGAGTAGAAGAGCGGTACCTCTATCTGGTGCCCTAA
SapI-R	ATTAATCCATGGCGATGAAGAGCGGATCCCATGCTACTCCTACCA
A-BsaI-F	ATTAATGGTCTCaTCGAGCGCAATTAACCCTCACTAAAGGG
A-BsaI-R	ATTAATGGTCTCaACCTCGTAATACGACTCACTATAGGGCG
B-BsaI-F	ATTAATGGTCTCaAGGTGCGCAATTAACCCTCACTAAAGGG
B-BsaI-R	ATTAATGGTCTCaGTTCCGTAATACGACTCACTATAGGGCG
C-BsaI-F	ATTAATGGTCTCaGAACGCGCAATTAACCCTCACTAAAGGG
C-BsaI-R	ATTAATGGTCTCaGGCCCGTAATACGACTCACTATAGGGCG
BCD-F	ATTAATGGATCCGGGCCCAAGTTCACTTAAAAAGGAGATCAACAATGA AAGCAATTTTCGTACTGAAACATCTTAATC
BCD2-Fbp-F	GAAAGCAATTTTCGTACTGAAACATCTTAATCATGCTAAGGAGGTTTT CTAATGAACCTAAAGAACCCCGAAACGC
BCD21-Fbp-F	GAAAGCAATTTTCGTACTGAAACATCTTAATCATGCGAGGGATGGTTT CTAATGAACCTAAAGAACCCCGAAACGC
BCD8-Fbp-F	GAAAGCAATTTTCGTACTGAAACATCTTAATCATGCATCGGACCGTTTC TAATGAACCTAAAGAACCCCGAAACGC
Fbp-R	ATTAATGCGGCCGCTTACTATTTGTCATCGTCATCTTTATAATCGGTCG CGGTGGTGTAGTCAACC
BCD2-AcnR-F	GAAAGCAATTTTCGTACTGAAACATCTTAATCATGCTAAGGAGGTTTT CTAATGTCCGTAGCGGCAGGCGA
BCD21-AcnR-F	GAAAGCAATTTTCGTACTGAAACATCTTAATCATGCGAGGGATGGTTT CTAATGTCCGTAGCGGCAGGCGA
BCD8-AcnR-F	GAAAGCAATTTTCGTACTGAAACATCTTAATCATGCATCGGACCGTTTC TAATGTCCGTAGCGGCAGGCGA

AcnR-R	ATTAATGCGGCCGCTTACTATTTGTCATCGTCATCTTTATAATCGTCGC GTTTACGGACAGTTCCCTCG
BCD2-Mez-F	GAAAGCAATTTTCGTACTGAAACATCTTAATCATGCTAAGGAGGTTTT CTAATGACCATCGACCTGCAGCGTTC
BCD21-Mez-F	GAAAGCAATTTTCGTACTGAAACATCTTAATCATGCGAGGGATGGTTT CTAATGACCATCGACCTGCAGCGTTC
BCD8-Mez-F	GAAAGCAATTTTCGTACTGAAACATCTTAATCATGCATCGGACCGTTTC TAATGACCATCGACCTGCAGCGTTC
Mez-R	ATTAATGCGGCCGCTTACTATTTGTCATCGTCATCTTTATAATCAGCGT TTTGCGCTTCGGCGACGGCCTGGA
Fbp-SapI-F	ATTAATGCTCTTCAAGTGCGCAATTAACCCTCACTAAAGGG
Fbp-SapI-R	ATTAATGCTCTTCTCATCGTAATACGACTCACTATAGGGCG
AcnR-SapI-F	ATTAATGCTCTTCAATGGCGCAATTAACCCTCACTAAAGGG
AcnR-SapI-R	ATTAATGCTCTTCTATCCGTAATACGACTCACTATAGGGCG
Mez-SapI-F	ATTAATGCTCTTCAGATGCGCAATTAACCCTCACTAAAGGG
Mez-SapI-R	ATTAATGCTCTTCTCGACGTAATACGACTCACTATAGGGCG
